# Change in Insulin Sensitivity and Lipid Profile After Dopamine Agonist Therapy in Patients With Prolactinoma

**DOI:** 10.7759/cureus.17824

**Published:** 2021-09-08

**Authors:** Ghayyur Khalil, Feroz A Khan, Qazi M Jamal, Ayesha Saleem, Hassan Masroor, Kiran Abbas

**Affiliations:** 1 Department of Pulmonology, Glenfield General Hospital, Leicester, GBR; 2 Department of Orthopaedic Surgery, MMC General Hospital, Peshawar, PAK; 3 Department of Paediatrics, Qazi Hussain Medical Complex, Nowshera, PAK; 4 Department of General Surgery, Hayatabad Medical Complex Peshawar, Peshawar, PAK; 5 Department of Psychiatry, Psychiatric Care Associates, Peshawar, PAK; 6 Department of Medicine, Jinnah Postgraduate Medical Centre, Karachi, PAK

**Keywords:** dopamine agonist, prolactinoma, anterior pituitary, prolactin, insulin, diabetes mellitus type 2, hdl, ldl

## Abstract

Background

Prolactinomas are prolactin(PRL)-secreting neoplastic lesions that can lead to metabolic disturbances and insulin resistance. We aimed to find the change in insulin sensitivity and lipid profile in prolactinoma patients after dopamine therapy.

Methodology

A prospective observational study was conducted at the Hayatabad Medical Complex, Peshawar between June 2019 to July 2020. All patients with newly diagnosed prolactinoma were eligible to partake in the study. Individuals with multiple hormone-producing pituitary tumors, hyperprolactinemia secondary to other causes than tumors, and those taking medication for hypercholesterolemia were excluded. Diagnosis of prolactinoma was established on the basis of elevated PRL levels on at least two occasions three days apart in addition to an MRI detecting prolactinoma on the hypothalamic-pituitary area. The mean dose for dopamine agonist was 5.8 ± 4.1 mg per day with a range between 1.25 to 15 mg. To assess the change in insulin resistance, the homeostatic model assessment insulin resistance (HOMA-IR) was computed. All data were recorded in a predefined proforma.

Results

The difference between pre- and post-treatment values for mean PRL levels was statistically significant (p<0.0001). There was a significant association between dopamine agonist treatment and the BMI (pre- vs. post-treatment; 28.9 ± 4.28 vs. 24.53 ± 2.2, p<0.0001). Both low-density lipoprotein (LDL)-cholesterol and HOMA-IR scores were significantly lower in the post-treatment group as compared to the pre-treatment group (p<0.0001).

Conclusion

The present study indicated that dopamine agonist therapy was effective not only in lowering the serum PRL levels but also improved insulin sensitivity and decreased lipid metabolism, resulting in improved BMI. Further studies should explore the long-term side effects of dopamine agonists on patients with prolactinoma.

## Introduction

The anterior pituitary gland produces a polypeptide hormone called prolactin (PRL) [1]. PRL plays a significant role in the development of mammary glands in pregnant women and stimulates lactation [2].

A type of tumor that grows in the pituitary gland, known as prolactinoma, secretes the hormone prolactin. Prolactinomas contribute to 40% of all tumors of the pituitary. The global prevalence of prolactinoma is approximately 10% in males and 30% in females. Prolactinoma mostly occurs in females in the age group of 20 to 40 years [3]. 

The presenting symptoms of prolactinoma include amenorrhea, galactorrhea, infertility, and headache [2]. A strong association has been found between obesity and the development of prolactinoma [3-6]. Other associations of prolactinoma have been found with metabolic syndrome and insulin resistance [6,7].

The neurotransmitter called dopamine plays a significant role in energy expenditure as well as limits food intake and lactotroph function via dopamine D2 receptors [8]. Thus, dopamine agonist drugs are considered as the first-line treatment for patients who are suffering from prolactinoma but often result in weight loss as an adverse effect [8,9].

Hyperprolactinemia is strongly associated with obesity, insulin resistance, raised inflammatory markers, and endothelial dysfunction [4-6]. A significant improvement has been seen in patients with prolactinoma who are treated with dopamine, by a mass improvement in metabolic status [10]. 

Even though the relationship between obesity, BMI, and prolactinoma has been extensively explored, there are little to no studies conducted in the local population on the effect of dopamine agonist therapy on the Insulin sensitivity and lipid profile in prolactinoma patients. Therefore, the authors conducted the current study on a cohort of patients presented at the Hayatabad Medical Complex, Peshawar for the management of prolactinoma. 

## Materials and methods

A prospective observational study was conducted at Hayatabad Medical Complex, Peshawar, between June 2019 to July 2020. Ethical approval was obtained from the institutional review board committee prior to the study (Reference # F2-61-IRB-2019-GENL/24512/JPMC). All patients with newly diagnosed prolactinoma were eligible to partake in the study. Informed consent was obtained from all patient participants after the objectives and benefits of the study were explained. Individuals with multiple hormone-producing pituitary tumors, hyperprolactinemia secondary to other causes than tumors, and those taking medication for hypercholesterolemia were excluded from the study.

Diagnosis of prolactinoma was established on the basis of elevated PRL levels on at least two occasions, three days apart, in addition to MRI detecting prolactinoma on the hypothalamic-pituitary area. 

Sociodemographic and clinical characteristics of the study participants were documented in a predefined proforma. A detailed gynecological history was taken from all women participants. All patients were married. All women were premenopausal at the time of the study. Six women were on hormonal contraceptives, which were not discontinued through the study. Five women had secondary amenorrhea.

All participants were evaluated at baseline and after three months of dopamine agonist treatment at the outpatient set-up. The mean dose for dopamine agonist was 5.8 ± 4.1 mg per day with a range of 1.25-15 mg. For all patients, lipid profiles including serum cholesterol, triglycerides, high-density lipoprotein (HDL) and low-density lipoprotein (LDL) cholesterol, hemoglobin A1c (HbA1c), fasting blood glucose levels were advised and documented at each follow-up. Normal PRL levels for men were taken to be <20 ng/dl and < 25 ng/dl for women [11]. To assess the change in insulin resistance, HOMA-IR was computed by using the formula below:

(Fasting plasma glucose (mmol/l) X Fasting serum insulin (mU/l)) / 22.5 

The value of HOMA-IR is inversely related to insulin sensitivity. The higher the HOMA-IR score, the lower the insulin sensitivity, hence, indicating higher resistance. All data were analyzed using the IBM SPSS Statistics for Windows, Version 26.0 (Released 2019, IBM Corp, Armonk, NY). A paired sample t-test was applied to find out the association of dopamine agonist treatment with a decrease in PRL, lactate dehydrogenase (LDH), and BMI at three months of treatment. A p-value of less than .05 was set as the cut-off value for statistical significance. 

## Results

A total of 32 patients completed the study. There were 18 men and 14 women. The mean age at the time of diagnosis was 36 ± 9.54 years. Patients' sociodemographic and baseline parameters are presented in Table 1.

**Table 1:** Baseline Characteristics of Patients

Baseline Variables	n (%)	Mean (SD)
Age at diagnosis in years		36.09 ± 9.54
Gender		
Male	18 (56.25%)	
Female	14 (43.75%)	
Median serum prolactin at baseline in ug/L		611.93 ± 116.79
Mean BMI in kg/m2		28.91 ± 4.28
Size of tumor		
Microadenoma	17 (53.13%)	
Macroadenoma	11 (34.38%)	
Giant macroadenoma	4 (12.50%)	
Clinical presentation		
Headache	26 (81.25%)	
Amenorrhea	16 (50%)	
Galactorrhea	19 (59.38%)	
Infertility	13 (40.63%)	
Erectile dysfunction (men only)	7 (21.88%)	
Decreased libido	5 (15.63%)	
Gynecomastia	5 (15.63%)	

It was found that the difference between pre- and post-treatment values for mean PRL levels was extremely statistically significant (p<0.0001). There was a significant association between dopamine agonist treatment and the BMI (pre- vs. post-treatment; 28.9 ± 4.28 vs. 24.53 ± 2.2, p<0.0001). Both LDL-cholesterol and HOMA-IR scores were significantly lower in the post-treatment group as compared to the pre-treatment group (Table 2). 

**Table 2:** Patient Characteristics With Prolactinoma Pre- and Post-treatment With Dopamine Agonists

Parameters	Pre-treatment	Post-treatment	P-value
Mean BMI in kg/m2	28.9 ± 4.28	24.53 ± 2.2	<0.0001
Mean prolactin levels in μg/dl	611.93 ± 116.79	24.40 ± 1.62	<0.0001
Mean LDL-cholesterol	126.96 ± 18.66	92.93 ± 5.57	<0.0001
HOMA-IR	1.34 ± 0.17	0.94 ± 0.13	<0.0001

## Discussion

Our study revealed that the use of dopamine agonists in patients resulted in a reduction of levels of LDL following three months of therapy. Additionally, therapy led to an increase in insulin sensitivity in patients with prolactinoma, with significant reductions in the HOMA-IR score. A similar study conducted by Binart et al. found that dopamine agonist drugs caused a moderate decrease in LDL levels. The study reported a reduction in BMI and patient weight after initiating therapy. It concluded that the levels of HOMA-IR and fasting glucose following dopamine agonist therapy were significantly reduced in patients [12]. Previously it was shown that there is a positive association between PRL and obesity [13,14]. The results also highlighted the effect of PRL on adipocytes and lipid metabolism, causing a reduction in lipid levels. A possible reason for this is the presence of PRL receptors on the surface of adipose cells. 

Santos et al., failed to find an association between dopamine agonist therapy and reduced BMI [15]. It may be possible that longer treatment is essential to see changes in BMI with dopamine agonist therapy. Another reason for the lack of association may be due to the differences in the drug used in contrast with prior studies that confirmed alterations in BMI following therapy [16].

In conclusion, marked metabolic changes were seen in patients following dopamine agonist therapy. The metabolic alterations seen in patients of prolactinoma receiving treatment are complex and depend on multiple factors. Our results were based on a small group of patients. It is therefore recommended that large prospective studies are conducted, which would also assess the metabolic profile of patients with asymptomatic hyperprolactinemia who are receiving no therapy or have discontinued treatment with dopamine agonists. 

The study could further be enhanced by acquiring long-term data for a better evaluation of the therapeutic efficacy of dopamine agonists. Due to limited resources, patient follow-up at longer intervals was not possible. Thus, data regarding the long-term implications of dopamine agonist therapy could not be collected. 

## Conclusions

The present study indicated that dopamine agonist therapy was effective not only in lowering the serum prolactin levels but also improved insulin sensitivity, decreased lipid metabolism, resulting in improved BMI. Future studies should explore the long-term side effects of dopamine agonists reported by patients with prolactinoma.

Furthermore, large-scale studies should be conducted to determine the risk factors associated with non-compliance and resistance associated with dopamine agonist treatment among patients with prolactinoma. 
